# Movement Recognition Technology as a Method of Assessing Spontaneous General Movements in High Risk Infants

**DOI:** 10.3389/fneur.2014.00284

**Published:** 2015-01-09

**Authors:** Claire Marcroft, Aftab Khan, Nicholas D. Embleton, Michael Trenell, Thomas Plötz

**Affiliations:** ^1^Neonatal Service, Royal Victoria Infirmary (RVI), Newcastle upon Tyne Hospitals NHS Foundation Trust, Newcastle upon Tyne, UK; ^2^MoveLab, The Medical School, Newcastle University, Newcastle upon Tyne, UK; ^3^Culture Lab, School of Computing Science, Newcastle University, Newcastle upon Tyne, UK

**Keywords:** preterm birth, cerebral palsy, neuro-motor assessment, general movement assessment, movement recognition

## Abstract

Preterm birth is associated with increased risks of neurological and motor impairments such as cerebral palsy. The risks are highest in those born at the lowest gestations. Early identification of those most at risk is challenging meaning that a critical window of opportunity to improve outcomes through therapy-based interventions may be missed. Clinically, the assessment of spontaneous general movements is an important tool, which can be used for the prediction of movement impairments in high risk infants. Movement recognition aims to capture and analyze relevant limb movements through computerized approaches focusing on continuous, objective, and quantitative assessment. Different methods of recording and analyzing infant movements have recently been explored in high risk infants. These range from camera-based solutions to body-worn miniaturized movement sensors used to record continuous time-series data that represent the dynamics of limb movements. Various machine learning methods have been developed and applied to the analysis of the recorded movement data. This analysis has focused on the detection and classification of atypical spontaneous general movements. This article aims to identify recent translational studies using movement recognition technology as a method of assessing movement in high risk infants. The application of this technology within pediatric practice represents a growing area of inter-disciplinary collaboration, which may lead to a greater understanding of the development of the nervous system in infants at high risk of motor impairment.

## Introduction

Each year more than 15 million babies worldwide are born preterm (before 37 weeks gestational age) and the number of cases continues to rise (1). Infants born preterm are at higher risk of developing motor impairment than infants born at term (2). Morbidity is inversely correlated to gestational age meaning that those born extremely preterm (<28 weeks gestation) are most at risk (3). Cerebral palsy (CP) is a common motor impairment (3) for high risk infants (such as those born preterm) and these infants are also at high risk of developmental delay and other motor coordination disorders (4).

There are currently no standardized clinical guidelines for the prediction of motor impairment in high risk infants and the identification of those at highest risk typically involves the integration of clinical history, neuroimaging results, different clinical assessments, and experience of health care professionals. The assessment of spontaneous general movements is an important tool, which can be used for the prediction of movement impairments in high risk infants (5).

Movement recognition aims to capture and analyze relevant limb movements through computerized approaches focusing on continuous, objective, and quantitative assessment. Different methods of recording and analyzing infant movements have recently been explored. Camera-based solutions (6–10) and body-worn miniaturized movement sensors (11–14) have been applied in order to record continuous time-series data that represent the dynamics of limb movements. Various machine learning methods have been developed to analyze the recorded movement data. This has specifically focused on the detection and classification of atypical spontaneous general movements.

The aim of this article is to briefly summarize the current most evidence based clinical approach to observational movement assessment in high risk infants (Prechtl’s General Movements Assessment) and identify the current studies, which have applied a variety of automated movement recognition technologies to assess infant movement.

## Clinical Prediction of Cerebral Palsy Using Prechtl’s General Movements Assessment

Infants learn how their bodies move and interact with the environment in early infancy (15). This is achieved through the development of spontaneous movements into goal directed movements through exploration and problem solving (15). Consequently, the development of spontaneous movements in early infancy is a high predictor for later movement (and also cognitive) performance (4, 16).

The development of spontaneous movements in infants has been studied and described in detail by Heinze Prechtl and colleagues (17). Prechtl’s general movements assessment is currently the clinical assessment that can most reliably predict CP in high risk infants with a reported sensitivity of 98% (95% confidence interval, CI 74–100%) and specificity of 91% (95% CI 83–93%) (5).

The application of this assessment clinically involves the evaluation of the qualities of the spontaneous general movements through Gestalt perception of the observer (18). A video recording is taken of the infant’s spontaneous general movements, which is then assessed during playback of the video recording at normal speed and in addition, a higher speed may be used to identify the presence of movement stereotypies (18).

In typically developing infants, spontaneous movements are characterized by large variation (18, 19). Writhing movements (observed between 36 weeks and 2 months post term) are performed with moderate amplitude and speed and are characterized by high complexity and large variation in relation to amplitude, velocity, and acceleration (18). Between 2 and 5 months of age, fidgety movements become apparent: these show smaller amplitudes of circular shape, lower speed, and a higher variability in acceleration (18).

Atypical motor development is characterized by limited variation and limited variability in generalized movements (18). In particular, the presence of cramped synchronized general movements (CSGMs) during preterm and term age and the absence of fidgety movements at 3–5 months are strong predictors for later CP diagnosis (20, 21). CSGM’s are atypical and lack fluency, variation, and complexity and are also stereotyped in nature (limb and trunk muscles contract and relax nearly simultaneously) (18, 21).

The challenges of applying the GM assessment in practice relate to the availability of appropriately trained and skilled clinicians. Considerable training is required for an assessor to become reliable enough to make an accurate evaluation. The assessment is susceptible to observer fatigue (21) and is dependent on the behavioral state of the infant during recording (ideally an infant should be in an alert, awake state). By the time, a single GM assessment is most accurate in predicting CP (3–5 months) (5) an opportunity to influence the nervous system at an earlier stage of development may have been missed.

Despite good levels of inter-observer reliability with the GM assessment (22), there will always remain a degree of subjectivity in interpretation. In common with all techniques involving interpretation by a skilled and experienced observer (including for example, ultrasound scan interpretation), experience of interpretation will improve with time. Despite this, it is not possible to determine the nature and extent of any subjectivity; however, the lack of widespread adoption among clinical teams may suggest there is a concern.

These challenges in the early detection of motor impairments in high risk infants have led to an increasing interest in the use of automated movement recognition technologies being applied in this clinical area.

## Automated Movement Recognition for Clinical Movement Assessment

The use of automated movement recognition technology has been explored in many different clinical conditions such as dementia (23), Parkinson’s disease (24), and Autism (25, 26). Many of these applications have produced promising results regarding the overall potential for continuous and longitudinal assessments in both clinical and home environments (27).

Movement recognition in clinical applications aims at the automated detection, classification, and assessment of the quality of limb movements focusing on indications for abnormalities. Capturing limb movements can be based on either indirect or direct sensing (28). Indirect sensing utilizes devices that are integrated into the assessment environment, such as video cameras or 3D motion capture facilities (29) and direct sensing records movement data through sensors that are worn by the patient, i.e., miniaturized devices attached to the limbs (11, 30, 31). Both methods capture movement as time-series data, i.e., sequences of sensor readings. Depending on the sensing modality applied, this data exhibit differences in temporal and spatial resolution, which requires adaptations of the analysis methods. Typical methods for automated data analysis include sequential probabilistic modeling, e.g., using Markovian approaches (32), statistical methods (11, 14) to model specific gestures of interest (12) or holistic monitoring of general movements (27).

There are considerable challenges in utilizing this technology in the neonatal environment, in particular, the size, fragility, and vulnerability of infants needs to be taken into account. Furthermore, the automated movement assessment must be sensitive and reliable enough to detect subtle changes in movements, which are the basis for clinical diagnosis.

Table 1 summarizes some of the advantages and disadvantages associated with the two sensing modalities, i.e., direct sensing, where movements are captured using hardware directly attached to the subject; and indirect sensing, where hardware is placed in the assessment environment.

**Table 1:** **Brief overview of some of the advantages and disadvantages associated with various sensing modalities in the context of recording general movements in preterm infants**.

		Advantages	Disadvantages
Indirect sensing	Video cameras (33, 36, 63)	1. Easy to understand	1. Computationally expensive analysis
		2. High spatial resolution	2. Privacy concerns
		3. High context information	3. Large disk space requirements
		4. Portable	4. Generally low temporal resolution
		5. High availability	5. Occlusion issues
	3D motion capture (29, 37)	1. High spatial resolution	1. High costs
		2. Depth information	2. Computationally very expensive analysis
		3. Accurate motion capture	3. Privacy concerns
		4. High reliability	4. Very large disk space requirements
		5. High temporal resolution possible	5. Large physical space requirement
		6. Secondary movement analysis possible (such as force and weight exchange)	6. Markers needed for motion capture
			7. Occlusion issues
	Microsoft kinect (35, 47, 62)	1. High spatial resolution	1. Not suitable for infants (<4 years)
		2. Depth information	2. Occlusion issues
		3. Low-cost	3. Low temporal resolution
		4. Marker-less motion capture	4. Limited field of view
Direct sensing	Wearable movement sensors (11–13, 34, 39–43, 45, 64)	1. High temporal resolution	1. Low spatial resolution
		2. Low-cost	2. Occasional data losses (wireless)
		3. Energy efficient	3. Limited battery life (wireless/real-time)
		4. Privacy preserving	4. Difficulty in consistent positioning
		5. Small physical size	5. Comfort issues
		6. Good battery life (embedded)	6. Relative movement capture only
		7. High availability (e.g., mobile phones)	
		8. Actigraphs: sleep/wake patterns	
	Magnet tracking system (31, 46, 66)	1. High temporal resolution	1. High costs compared with accelerometers
		2. Very high accuracy	2. Computationally very expensive analysis
		3. Metal tolerant	3. Complex setup
		4. No line of sight occlusions	4. Magnetic and electrical interference issues

Figure S1 in Supplementary Material gives an overview of the general approach to automated gesture recognition for clinical movement assessment. Sensory data for capturing movements using one or more of the sensing techniques (summarized in Table 1) are usually *pre-processed* [see in Ref. (40, 48, 49)] for wearable sensors and (50, 51) for vision-based systems followed by movement *segmentation*, i.e., parts from the recorded data are partitioned that might contain important movement information [see in Ref. (26) for automatic segmentation]. This is usually followed by *feature extraction* used to represent large amounts of sensing data in a reduced fashion [e.g., (52); see also Ref. (53) for a generic feature learning approach]. Finally, *classification* is performed to identify (or predict) different types of movements. Various classification frameworks have been reported in the context of wearable/environmental sensors such as (54–56) and vision-based systems for activity recognition (57–59). A supervised classification framework also requires some form of ground truth labeling. These labels are used for training a machine learning classifier [some issues related to ground truth annotation have been addressed in Ref. (48, 60); automated vision-based annotation systems have also been explored in Ref. (59, 61)].

In the following sections, we identify and classify existing systems for gesture recognition based automated movement assessment in preterm infants.

## Automated Movement Recognition for Clinical Movement Assessment in High Risk Infants

### Video-based assessment

Existing video-based movement assessment systems for infants can be categorized into: (i) using three dimensional (3D) motion capture systems; and (ii) using traditional color cameras. Motion capture based systems require special markers to be attached to the limbs being tracked. High-end cameras typically provide very high 3D tracking accuracy and resolution (both spatially and temporally; Figure S1 in Supplementary Material), but at a considerable price and setup effort. These systems are most commonly seen in the research setting and due to practical limitations are not easily adaptable to the clinical environment.

Meinecke and colleagues applied a motion capture system to objectively measure the spontaneous movements of infants during the first months of life (29). Fifty-three movement-based parameters were automatically extracted from motion tracks followed by cluster analysis based on Euclidian distances that selected eight of the parameters. These were able to delineate between healthy and at risk infants. Classification was performed using quadratic discriminant analysis (sensitivity 1, specificity 0.7) (see Table 2). With a similar setup Kanemaru and co-workers analyzed spontaneous movements in infants aiming to investigate the relationship between spontaneous movements and the development of CP at 3 years of age. The authors found that the jerkiness in spontaneous movements at term age (defined as the time integral of the square of the magnitude of jerks per unit movement distance) was higher in infants who developed CP (8–10).

**Table 2:** **Identification of different automated gesture recognition systems applied to objectively measure movement in infants**.

Maintainer	Movements/predictions	Clinical outcome	Sensing technology	Data analysis		Dataset/study	Results/findings^a^ (sensitivity, specificity)	Reference
				Data preprocessing	Classification method	
RWTH Aachen, Germany	GM/	24 months (CP/no CP)	Accelerometry	32 features using velocity + acceleration (73, 74)	Decision trees	23 Infants	(1.00, 0.86)	(45)
	1. Healthy		19 Healthy	
	2. At risk		4 High risk	
Seirei Christopher University, Japan	SM/	Nil doc	Accelerometry	MEM + FNN + MLE + Amplitude adjusted Fourier Transform	Mann–Whitney *U test + Student’s t-test*	14 Infants	With BI – high dimensional, unstable, and unpredictable movement	(13)
	1. With BI		7 High risk	
	2. Without BI		7 Low risk	
UC Irvine, USA	GM/	Nil doc	Accelerometry	Statistical features using acceleration including; mean, standard deviation, min, max, products, *z-value*	DT + SVM + DBN-RF	10 Infants	(0.103, 0.939) + (0.069, 0.964) + (0.498, 0.764	(11)
	1. CSGM present					6 CSGM present	
	2. CSGM absent					4 CSGM absent	
University Children’s Hospital Bern, Switzerland	SM/	Nil doc	Accelerometry	Detrended fluctuation analysis (DFA)	*t-test statistical test + Linear regression + generalized least squares regression*	22 Healthy infants	Correlation study	(14)
	1. Only healthy							
UC Irvine, USA	GM/	Nil doc	Accelerometry	Several basic motion features using acceleration + mean, max, min, SD, *z-score*	RF + Boosted NB + SVM + EC/DBN	10 Infants	(0.72, 0.57)	(12)
	1. CSGM present	
	2. CSGM absent	
NTNU, Trondheim, Norway	GM/(Visualization)	24 months CP/no CP	Accelerometry + Computer vision	Periodicity + PCA	n/a	14 Patients 4 FM types	n/a (visualization only)	(69)
University of Heidelburg, Germany	GM/	Nil (31, 52)	Magnet tracking system + computer vision	Stereotypy score, Periodic and Torpid leg movements	*t-test*	67 Infants	(0.90, 0.95)	(31, 46, 66)
	1. CP	24 mon (46)				49 High risk	
	2. Non-CP					18 Low risk	
NTNU, Trondheim, Norway	GM/	Not specified	Accelerometry + Computer vision	Skewness, cross-correlation, areas calculated using the moving average, Periodicity, PCA, AR + Linear Separability (scatter matrix), and Clustering analysis	1. LDA	81 Infants	(0.86, 0.90)	(70)
	1. Healthy				2. QDA			
	2. At risk							
University of Tokyo, Japan	SM/	3 years Dev Delay	3D Motion capture	6 Movement indices (using Frame-DIAS; DHK, Japan) including Jerk index (time integral of the square of the magnitude of jerks per unit movement distance)	Kruskal–Wallis test + Fisher’s exact test + Mann–Whitney *U test*	145 Infants 16 CP 129 Normal	Significantly higher jerk index in CP	(8–10)
	2. Non-CP							
	2. Non-CP							
St. Olav University Hospital, Trondheim, Norway	GM/	5 years (CP/no CP)	Computer Vision	Quantity of motion, Centroid of motion, Variability of velocity and acceleration, CP predictor feature	*t-test statistical test + Mann–Whitney U test + Logistic regression*	30 High risk infants	(0.85, 0.88)	(7)
	1. CP							
	2. Non-CP							
St. Olav University Hospital, Trondheim, Norway	GM/	Nil doc	Computer Vision	Quantity of motion, Centroid of motion, Variability of velocity and acceleration	Threshold analysis	82 Infants50 Low risk32 High risk	(0.815, 0.70)	(6)
	1. FM present							
	2. FM absent							
RWTH Aachen, Germany	GM/	Nil doc	3D Motion capture	Skewness, cross-correlation, area outside the SD of moving average, Area differing from moving average + Cluster analysis with Euclidian distances	QDA	22 Infants 15 Healthy 7 Affected	(1.00, 0.70)	(29)
	1. Healthy							
	2. At risk							

*GM, general movements; SM, spontaneous movements; CSGM, cramped synchronized GM; CP, cerebral palsy; BI, brain injury; PCA, principal component analysis; SD, standard deviation; DT, decision tree; NB, naïve Bayes; MEM, maximum entropy method; FNN, false nearest neighbors; MLE, maximal Lyapunov exponent; AR, auto-regression; SVM, support vector machine; QDA, quadratic discriminant analysis; LDA, linear discriminant analysis; EC/DBN, Erlang-Cox/dynamic Bayesian network*.

*^a^Predictive values of the assessment methods listed in the table need to be interpreted with caution as the number of infants included in the studies are mostly low*.

Despite the popularity of consumer 3D cameras such as Microsoft’s Kinect (47), these have not yet been used extensively for movement analysis in infants. This is in stark contrast to other clinical applications (35, 62). Although having great potential for general movement analysis, Kinect’s capabilities are not largely explored with regards to assessments of preterm infants. This could be because the provided human tracking system in Kinect, necessary for detailed analysis, is recommended for tracking humans who are at least 4 years old and therefore considered unsuitable for use with infants.

Standard video cameras such as regular web-cams, RGB cameras on tripods, and even video-enabled baby monitors (36) have also been used for marker-less capturing of infants’ body movements. These systems offer a more reasonable priced alternative to 3D motion capture system and in addition come with substantially less set up effort, which enables applications beyond research and clinical settings, allowing for continuous and more detailed analysis in natural (home) environments. Typically, these recording setups come with lower spatial and temporal resolution, which limits the level of detail of the analysis. Additionally, marker-less tracking is less accurate than professional motion capture (37), which may be problematic when attempting to identify more subtle differences in movement patterns. An example would be in identification of the differences between CSGMs and poor repertoire general movements. CSGMs are described as monotonous and rigid with the limb and trunk muscles contracting and relaxing simultaneously (33) and poor repertoire general movements are monotonous, with the amplitude, speed, and intensity lacking normal variability (33). While both of these movement patterns are identified as being atypical (18, 33), CSGMs can also be most easily recognized by human gestalt perception and also have a much higher predictive value for motor impairment (33). Therefore, identification of any difference between the two atypical patterns is clinically highly relevant.

Adde and colleagues have developed an advanced video-based analysis system for quantitative and qualitative assessments of infants general movements (63). Utilizing standard RGB video cameras and an analysis method that uses so-called “motion-grams” for quantifying changes in the infant’s movements, the general movements toolbox (GMT) is able to detect fidgety movements as described in the GM assessment (18). This clinical toolbox may be useful in the early diagnosis and/or risk stratification of infants at high risk of developing CP. The first studies using GMT on a small number of high risk infants (*n* = 30) report promising results (sensitivity 85% and specificity 88%) with respectable follow-up data past 4 years of age (7). This suggests that the application of the GMT as a method for prediction of neurological impairment may be straightforward, cost-effective, and feasible for use in clinical practice but will require further systematic validation.

### Assessment through direct movement sensing

While indirect sensing settings require external tracking equipment, body-worn accelerometers have recently been successfully applied and remain popular in clinical studies. In particular, the proliferation of affordable, reliable, and miniaturized sensing facilities in combination with sophisticated data analysis techniques has allowed for automation of movement assessment in small infants. Accelerometers are sensors, which measure inertial forces in one or three spatial axes, resulting in high-resolution time-series data that represent the dynamics of the acceleration of the sensor during movements (34). Specifically, they have been used to measure physical activity levels in children (34), sleep/wake cycles, and the physiology of swallowing in infants (38, 64). These applications are in line with a large body of research that focuses on objective assessments of movement related parameters in a multitude of health-related scenarios, including gait analysis (39) sport activity (40), rehabilitation monitoring (41, 42), quantification of disease progression, and investigation of effectiveness of therapy interventions (43).

Even though reduced frequency and quantity of limb movements in infants have recently been identified as early predictors for developmental delay (9), it is widely accepted that in infants with a suspected diagnosis of CP, the general movements change in quality rather than quantity (44, 65). An automated analysis therefore needs to capture fine details of limb movements, for which video-based assessments are often limited.

Karch et al. developed an electromagnetic tracking system to undertake movement analysis (66). Small lightweight sensors (1.3 mm diameter) were attached to the infant’s limbs, which were then sensed using a commercial (external) tracking system that provided high accuracy and allowed for detailed analysis of joint flexion. Serving as proof of concept this tracking system has successfully been used for detecting anomalous, spontaneous limb movements in infants (31, 66). Phillippi et al. conducted a recent study using this work where it was found that the stereotypy score of arm movements could be used as a predictor for CP and stereotyped periodic leg movements predicted neurodevelopmental impairments (46).

A limited number of more recent studies have successfully applied accelerometers to preterm infants to measure spontaneous movements (13, 31, 45, 46) and also to create models for atypical movement patterns such as CSGMs (11, 12). While this is an evolving area of translational use of technology in the healthcare setting, there is limited data quantifying limb movements and also comparing the results to longer-term neurodevelopmental outcomes. Ohgi et al. used tri-axial accelerometry for a cross-sectional study that measured upper limb acceleration in a small number of preterm infants with and without brain injuries at 1 month corrected age (13). This was the first application of accelerometers in preterm infants in a clinical environment but the recording time was limited to 200 s due to the variability of the infant’s states. Statistical analysis confirmed that infants with brain injuries exhibited unstable and unpredictable spontaneous movements with larger dimensionality (details of the analysis techniques and statistical tests are provided in Table 2).

A group from University of California, Irvine have tested the use of wireless accelerometers in the neonatal unit and compared accelerometer data to the general movements assessment (11, 12). CSGMs are highly predictive of CP (21, 67) and the group investigated whether accelerometry, combined with machine learning techniques for automated data analysis, could accurately identify the components of these atypical movements. By means of this automated assessment, limb acceleration and correlation have been characterized successfully. Gravem et al. used various statistical classification techniques to correctly identify the presence of CSGM in 6 out of 10 infants (11). Reported accuracy varied between 70 and 90% (see Table 2 for details).

Heinze et al. used miniature accelerometers with the aim of developing a methodology to allow objective diagnosis of the development of movement disorders in preterm infants (45). The overall detection rate was >90% over three measurements and the timing of the data collection correlated with the characteristics of spontaneous movements as described in the general movements assessment (18). Nineteen healthy term infants and four “at risk” preterm infants were included in the study. Clinical and neurological examinations were undertaken and “at risk” infants were identified through abnormalities on computer tomography and/or follow-up to 2 years.

These direct sensing studies represent promising applications of technology to contribute to the identification of atypical movement patterns in preterm infants. However, it is important to take into consideration that only small numbers of preterm infants with atypical movements were included which do not allow for the calculation of sensitivity and specificity. Furthermore, the feasibility of applying these techniques to larger numbers of high risk infants in a clinical environment is difficult to establish and in comparison to applying video-based methods of assessment (including Prechtl’s GM assessment) may be more difficult to achieve due to the logistics of attaching direct sensors to small infants.

#### Hybrid systems

The main advantage of video-based movement analysis lies in the recording of spatial data in addition to temporal measurements, which gives direct access to, for example, posture-related information. In contrast, direct sensing approaches typically have a much higher temporal resolution allowing for more detailed assessments, which is beneficial for in-depth analysis of subtle changes that may precede the development of CP.

Some approaches have been developed that aim at combining both sensing methods in order to benefit from both spatial and high temporal resolution – at reasonable costs and minimum setup efforts for practical applications – for high-fidelity movement analysis (68). Berge and colleagues have proposed a software tool for GM representation and modeling called ENIGMA – enhanced interactive general movement assessment. ENIGMA provides a useful support tool for visualizing features of motion data in conjunction with video data for GM experts (69). Similarly, Rahmanpour et al. employ a hybrid sensing approach for the prediction of CP using an extensive analysis of movement-based features. The authors found that dynamic features are more indicative than the standard statistical features (70). Results from their analysis are shown in Table 2 with a detailed description of the employed features.

## Summary

Prediction of motor impairment (such as CP) in preterm infants is challenging, and ideally requires techniques that are both sensitive and specific. Due to the large number of complex factors affecting neurodevelopment, and the difficulty in assessing brain plasticity, predicting which children will develop CP on the basis of a single assessment will always be challenging (71). A multi-modal longitudinal approach including a combination of methods, e.g., neurological assessment, general movements’ assessment, and neuroimaging is likely to improve both positive and negative prediction. Early risk stratification and prediction has many benefits. It allows for early identification of those most likely to benefit from early intervention and targeting of resources and support for parents. Furthermore, access to health, social, and educational services is often dependent on a diagnosis (72).

Accurate, non-invasive assessments of sufficient sensitivity to identify longitudinal changes in movement patterns could hold considerable hope for the future. This mini-review has identified recent studies employing video-based assessment, assessment through direct movement sensing and hybrid systems. Specifically, the use of accelerometry and computer vision may offer clinically feasible and promising methods of objectively measuring quality and quantity of infant movement. The application of these technologies may prove to be useful not only in the prediction of infants at highest risk of motor impairment but also in the evaluation of therapies aiming to influence the developing brain.

## Conflict of Interest Statement

The Guest Associate Editor Gavin John Clowry declares that, despite being affiliated to the same institution as authors Claire Marcroft, Aftab Khan, Michael Trenell, and Thomas Plötz, the review process was handled objectively and no conflict of interest exists. The authors declare that the research was conducted in the absence of any commercial or financial relationships that could be construed as a potential conflict of interest.

## Supplementary Material

The Supplementary Material for this article can be found online at http://www.frontiersin.org/Journal/10.3389/fneur.2014.00284/abstract
