# Supplementary material for: Movement Recognition Technology as a Method of Assessing Spontaneous General Movements in High Risk Infants
Source: Front Neurol. 2015 Jan 9;5:284. doi: 10.3389/fneur.2014.00284 (PMC4288331; doi:10.3389/fneur.2014.00284)
Supplement: Supplementary file 1 [file Image_1.PDF]

Supplementary Material

Movement recognition technology as a method of assessing spontaneous general movements in high risk infants

Claire Marcroft*[1,2], Aftab Khan[3], Nicholas D Embleton[1,], Michael Trenell[2], Thomas Plötz[3]

[1] Newcastle upon Tyne Hospitals NHS Foundation Trust, Neonatal Service, ward 35, RVI, Newcastle upon Tyne, UK
[2] MoveLab, The Medical School, Newcastle University, Newcastle upon Tyne, NE2 4HH, UK
[3] Culture Lab, School of Computing Science, Newcastle University, Newcastle upon Tyne, NE1 7RU, UK

*** Correspondence:** Ms Claire Marcroft, Newcastle Neonatal Service, Special Care Baby Unit (ward 35), Royal Victoria Infirmary, Newcastle upon Tyne, NE1 4LP
c.marcroft@newcastle.ac.uk

1. Supplementary Figures

Figure S1: Overview of automated gesture recognition for clinical movement assessment showing video-based, accelerometer-based (courtesy of Fan 2012) (12), and 3D motion capturing systems (courtesy of Meinecke 2006) (29).

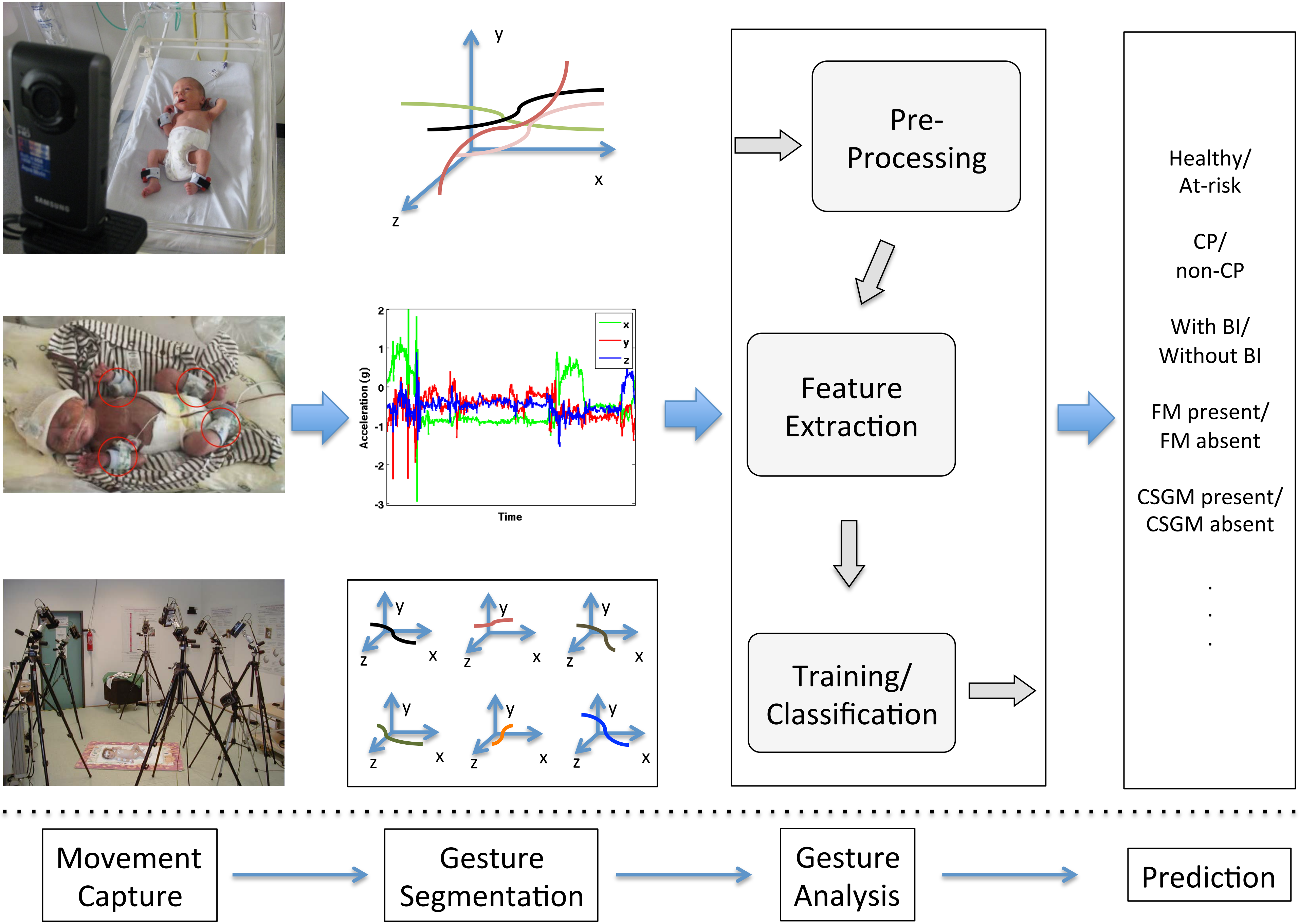

y
x
z
Acceleration (g)
Time
2
1
0
-1
-2
-3
x
y
z
Pre-
Processing
Feature
Extraction
Training/
Classification
Healthy/
At-risk
CP/
non-CP
With BI/
Without BI
FM present/
FM absent
CSGM present/
CSGM absent
Movement
Capture
Gesture
Segmentation
Gesture
Analysis
Prediction
